# Challenges for vaccination in the elderly

**DOI:** 10.1186/1742-4933-4-9

**Published:** 2007-12-11

**Authors:** Richard Aspinall, Giuseppe Del Giudice, Rita B Effros, Beatrix Grubeck-Loebenstein, Suryaprakash Sambhara

**Affiliations:** 1 Department of Immunology, Imperial College, London, UK; 2 Novartis Vaccines, Via Fiorentina 1, 53100 Siena, Italy; 3 Department of Pathology and Laboratory Medicine, David Geffen School of Medicine at UCLA, Los Angeles, USA; 4 Institute for Biomedical Aging Research, Austrian Academy of Sciences, Innsbruck, Austria; 5 Influenza Division, Centers for Disease Control and Prevention, Atlanta, USA

## Abstract

The increased susceptibility of the elderly to infection presents a major challenge to public health services. An aging immune system is well documented as the cause of increased infection rates in elderly people. Such immunosenescence is multi-factorial and incompletely understood. Immunosenescent changes include malfunctioning of innate immune system cellular receptors; involution of the thymus, with consequent reduction of the naïve T cell population; alteration of the T cell population composition; modified phenotypes of individual T cells; and replicative senescence of memory cells expressing naïve markers. Unfortunately, immunosenescence also renders vaccination less effective in the elderly. It is therefore important that the vaccines used against common but preventable diseases, such as influenza, are specifically enhanced to overcome the reduced immune responsiveness of this vulnerable population.

## 1. Introduction

The global population is aging and the percentage of the population that is elderly (≥60 years of age) now forms a larger proportion than ever before. Analysis undertaken by the United Nations has shown that the percentage of elderly people worldwide has increased from 8% in 1950 to 10% in 2000; this trend is expected to continue, with 21% of the population being elderly by 2050 [1].

The aging population presents a challenge for the public healthcare system, as the elderly suffer from more frequent and severe infections than younger people [2]. Furthermore, elderly people tend to experience poor outcomes from infections in comparison to the younger population. In particular, influenza is an example of a common infection which causes annual epidemics, and in the elderly, is associated with increased morbidity. Indeed, influenza is one of the ten major causes of death in the elderly [2].

One of the main reasons for the increase in infections observed in the elderly is believed to be immunosenescence [2]. This term was introduced by Dr. Roy Walford [3] and refers to the immune system's diminished function with age [4], which leads to a decline in the response to infection by both the innate and adaptive immune systems. This phenomenon, however, is not yet fully understood [5].

As the immune response in the elderly declines and the outcome of infection is often poor, prevention of infections becomes critically important [4]. Vaccination can protect the elderly against diseases such as influenza, and in this case is recommended by the World Health Organization [6] and other health authorities. However, immunosenescence also affects the response to immunisation, as shown by the reduced efficacy of annual influenza vaccination in the elderly, with an efficacy of 17–53% in the elderly, compared with 70–90% in healthy adults [7].

The effects of immunosenescence on the innate immune response, the generation of T cells, the adaptive immune response, and the response to vaccination are discussed here, highlighting the need for a better understanding of the effects of aging on the immune response and the development of more effective vaccines that target the elderly.

## 2. Aging changes innate immunity

Innate immunity is a key element of the immune response; it prevents the entry of pathogens into the body's tissues, rapidly removes microorganisms if they gain access into the tissues, and instructs the adaptive immune system to mount pathogen-specific humoral and cellular immune responses. The innate immune system includes several cellular components such as macrophages, natural killer cells, and neutrophils, which provide first-line defence against bacterial and viral infections [8]. The function of these cells declines with age, which may offer an explanation for the increased incidence of gastrointestinal and skin infections, as well as an increase in bacterial and viral pneumonias observed in the elderly. Immunosenescence of the innate immune system may also contribute to the reduced response to vaccination, such as for influenza; while influenza vaccination has 70–90% efficacy in healthy adults, this falls to 17–53% in the elderly [7]. This reduced efficacy is due to changes in the microenvironment leading to suboptimal stimulation of the adaptive immune system.

The innate immune system detects pathogens using pattern-recognition receptors, such as toll-like receptors (TLR), which recognise specific molecular patterns present on the surface of pathogens triggering a variety of signalling pathways. TLR are evolutionarily conserved molecules expressed on a variety of cells, such as macrophages, and form a large family of related molecules [8,9]. Interaction between a TLR and a pathogen stimulates the secretion of a wide range of antibacterial peptides that destroy the pathogen and trigger an inflammatory response [9]. Changes in the expression and function of TLR as a result of immunosenescence, leading to dysregulated pro-inflammatory cytokine and chemokine secretion, may explain why the elderly fail to exhibit classical symptoms of some infectious diseases. Furthermore, alteration in the functioning of TLR may interrupt instruction of the adaptive immune system, resulting in an inadequate response.

**Figure 1:**
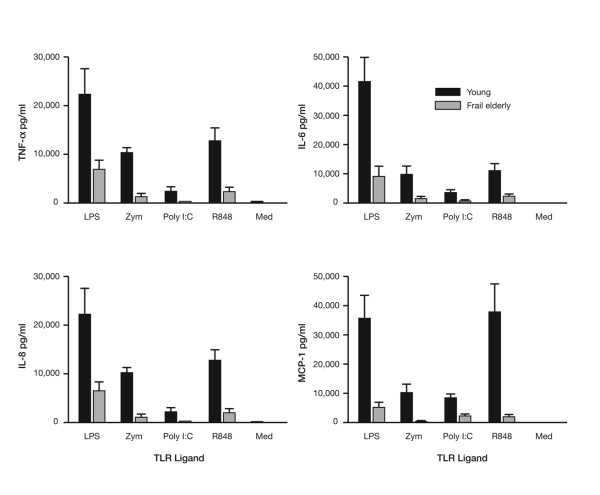
**Monocytes from frail elderly subjects secrete reduced levels of proinflammatory cytokines and chemokines in response to toll-like receptors (TLR) ligation**. TNF-α, tumour necrosis factor alpha; IL-6, interleukin-6; IL-8, interleukin-8; MCP-1, monocyte chemotactic protein-1; LPS, lipopolysaccharide; Zym, zymosan; Poly I:C, polyinosinic-polycytidylic acid; R848, resiquimod; Med, medium alone. Results are from 1 × 10^6 ^monocytes from nine young and nine elderly individuals stimulated with various TLR ligands and analyses of culture supernatants 48 hours post-stimulation. The error bars represent standard error of means.

Studies comparing macrophage TLR from young and old mice using an *in vitro *system have shown that expression and function of these receptors declines with age [8]. Furthermore, there is a decrease in the secretion of pro-inflammatory cytokines in aged mice, which is replicated in frail, elderly people (Figure 1) [10]. These findings demonstrate the impact of immunosenescence on the expression and function of TLR, resulting in changes which affect the innate immune response, which in turn affect the adaptive response. Therefore, modulation of the innate immune system either with TLR ligands or the products of TLR activation may enhance disease resistance, immune response and vaccine effectiveness in the elderly.

## 3. Immunosenescence affects the response to vaccines

In a young healthy adult (<30 years of age) there are approximately 3 × 10^11 ^T cells, of which 1–2% can be found within the blood, and up to 50% are within the antigen-naïve population. During a successful response, activation of these antigen-naïve T cells leads to their clonal expansion, the generation of effector cells and the subsequent reduction in the amount and source of the antigen. This is followed by a period of cell death since the immune system no longer requires large numbers of T cells bearing that specific antigen receptor; however, some cells remain and become memory T cells that subsequently enter the memory T cell pool. Repeated exposure of the immune system to a potential pathogen will be met by these memory T cells and will lead to a response that is more rapid and of greater magnitude than the response following the initial exposure. As there are few completely sterile environments, each of us is confronted on a daily basis with different organisms. Thus, our survival depends upon our immune system recognising and responding successfully to a broad range of potential pathogens. Provided these pathogens do not result in our death, our immunological memory should increase; indeed, analysis shows that aging is associated with an increase in the number of memory T cells. Theoretically then, with this increased memory T cell pool, we should be able to cope with more infections as we get older. Unfortunately this does not seem to be the case. Evidence from epidemiological, clinical and laboratory studies suggest an age-related defect in the immune system. The epidemiological evidence reveals that older individuals are often the first to be affected by new or emerging pathogens such as West Nile virus [11]. During an epidemic of West Nile virus in the United States in 2002, the majority of cases occurred in those over 50 years of age. The epidemic caused 4156 cases, of which 284 were fatal; the median age of the deceased was 78 years of age [12]. Clinicians recognise that in addition to this susceptibility to new pathogens, older individuals often have difficulties in dealing with pathogens which they have previously overcome, including the annual return of influenza. Although influenza infection is considered to be a self-limiting contagious viral-mediated disease of the respiratory tract, it is associated with considerable morbidity and mortality in the elderly. Those >65 years of age account for >90% of the deaths from influenza and are more likely to develop complications such as pneumonia following infection [13].

Such age-associated dysfunctions are preceded by a measurable decline in thymic export of αβ^+ ^T cells [14,15] to the naïve T cell pool, which declines with age due to the combination of the limited lifespan of naïve cells, reduced thymic function, and recruitment of naïve cells into activated and memory T cell pools. Homeostatic mechanisms, however, ensure that numbers within the total T cell pool are maintained through life within specific limits, so a decrease in naïve T cell numbers is matched by an increase in the number of memory T cells [16,17] and senescent T cells [18].

The age-associated alteration in the number of naïve T cells emerging from the thymus is thought to be caused by changes in the thymic microenvironment that prevent thymopoiesis. One element recently implicated in these changes is interleukin-7 (IL-7) [19]. IL-7 has a central role in the production of T cells. The receptor for IL-7, which comprises a common γ chain and an α chain, is expressed during the intrathymic T cell developmental pathway [20]. Interaction between IL-7 and its receptor at early stages of the T cell pathway has been reported to aid cell survival [21] and also act as a cofactor in recombination events [22]. At later thymocyte stages, this interaction may act to expand positively selected thymocytes [23]. An age-related reduction in production of IL-7 within the thymus [19,24] contributes to the reduced survival of thymocytes [25] and it is this reduction which produces the age-related decline both in thymic size and thymic output.

**Figure 2:**
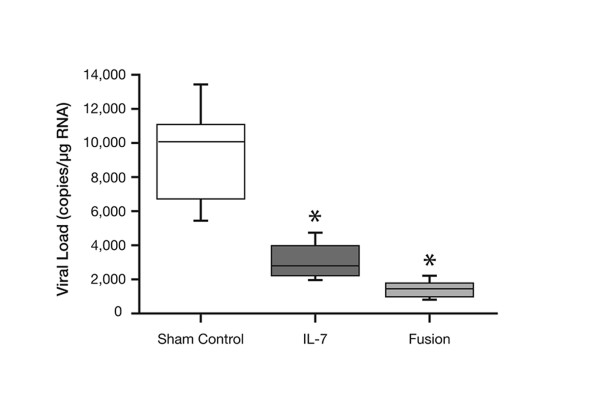
Influenza viral load in the lungs of 20 month old mice, 6 days after infection. Copyright 2005 The American Association of Immunologists, Inc. *P< 0.001 when compared with the control group. These mice had expression plasmids containing either the IL-7 gene, the fusion protein (the extracellular portion of the chemokine receptor CCR9 and interleukin-7) gene, or a sham control gene (the N-terminal portion of CCR9) inserted into their anterior tibial muscle approximately 2 months previously [26].

The decline in IL-7 expression levels makes it a target for therapeutic interventions to rejuvenate thymopoiesis in the elderly. Previous work has shown that IL-7 can reverse the atrophy of the thymus in old animals, ensuring increased thymic output to the peripheral T cell pool and improving immune responses [25]. The normal therapeutic approach has been to inject IL-7 subcutaneously so that it will diffuse through the organs and tissues of the body to reach its target organ. This approach is inefficient because of the low concentration of IL-7 that eventually reaches the thymus. As with most therapeutic agents, there is likely to be a threshold IL-7 concentration requirement, below which it has no effect. The approach taken to overcome this problem was to target IL-7 to the thymus by the creation of a fusion protein. The molecule CCL25 is produced in the thymus [26] and binds to the chemokine receptor CCR9 for which it is the only known ligand. A fusion protein between the extracellular portion of CCR9 and IL-7, when used as a therapeutic agent in old animals, results in the accumulation of the fusion protein in the thymus, the reversal of age-associated thymic atrophy, a significant increase in the production of new T cells and a significant improvement in antiviral responses in old animals [26]. An example of the effect of the fusion protein treatment is provided in Figure 2 which shows that mice treated with the fusion protein had a lower influenza viral load in their lungs, compared with sham-treated animals, following influenza infection.

IL-7 clearly has an important role in thymic functioning and these findings indicate that modulation of IL-7 may offer a target for increasing the response to vaccination in the elderly.

## 4. The challenges of immunity to infection in the elderly

The effects of immunosenescence on the thymus and resulting decline in the population of naïve T cells have already been mentioned. As the ratio of naïve T cells (i.e., cells that have not yet encountered a specific antigen) [27] to memory T cells falls, the repertoire of cells available to respond to challenges from novel pathogens shrinks [28,29]. Theoretically, this small population of cells should still proliferate and be functional, offering protection against new infections [27]. Furthermore, these cells could be specifically targeted by vaccines to stimulate specific and effective immune responses. However, evidence suggests that although these cells have a naïve phenotype they may also have aged.

**Figure 3:**
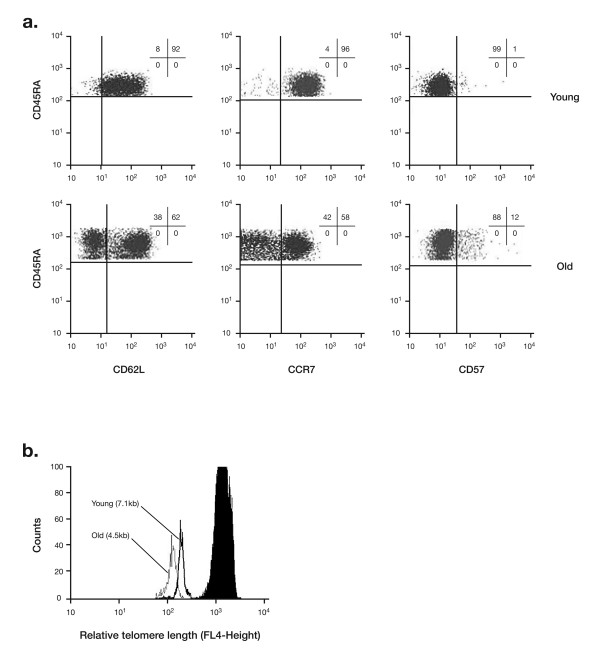
**CD8^+ ^CD45RA^+^CD28^+ ^T-cells from 5 young (<35 years of age) and 5 elderly (>65 years of age) donors**. **A) Expression of CD surface markers by fluorescent-activated cell sorting (FACS) analysis [30]**. Numbers in the graph indicate the percentage of cells in the respective quadrant. Ten identical experiments were performed. **B) Flow fluorescent *in situ *hybridisation (FISH) analysis of the relative telomere length [30]**. Filled black peak represents the tetraploid human T-cell leukemia cell line 1301 with a known telomere length of 25 kbp (internal standard). The figure shows one of ten identical experiments.

The phenotype and function of the remaining naïve T cell population in the elderly was evaluated and compared with those of younger people [27]. Fluorescent-activated cell sorting (FACS) analysis was used to detect surface markers that should be abundant in this population of cells such as the T cell homing receptors CD62L and CCR7. In addition, levels of CD57, the senescence marker, were measured, which was expected to be expressed at low levels. The results indicate that naïve T cells from the younger donors form a homogenous population, the majority of which express CD62L and CCR7, with very few cells expressing CD57 (Figure 3A) [30]. However, the naïve T cells from elderly donors formed a much more heterogeneous population. Approximately 40% of cells did not express the T cell homing receptors CD62L and CCR7, meaning these cells cannot migrate to peripheral lymph tissue. In addition, about 10% expressed the senescent marker CD57 (Figure 3A).

Further studies have shown that T cells from the elderly with a naïve phenotype (CD45 RA^+ ^CD28^+ ^as well as CD45 RA^+ ^CD28^+ ^CD62L^+^) had significantly shorter telomeres, a sign of reduced proliferative potential, than those from younger people (Figure 3B) [27,30]. In addition, the T cell receptor repertoire is restricted in all 24 Vβ families, suggestive of the presence of clonal expansions [27]. These changes were unexpected, since naïve T cells, irrespective of their source, should theoretically have long telomeres and should not have undergone clonal expansion.

In theory, increasing age would be predicted to be associated with a well-functioning memory T cell population that can respond to previously encountered pathogens. However, this is not always the case [31]. Among several factors that hamper the function of both naïve and memory T cells in the elderly, there is latent infection with cytomegalovirus (CMV) [32]. CMV seropositivity has been linked with changes of T cell phenotype and function similar to those observed in immunosenescence. In addition, this is thought to be one of the main factors stimulating oligoclonal expansion in CD8^+^ T cells. Overall, these findings suggest that phenotypic classification of naïve T cells may be inaccurate in the elderly. Furthermore, CMV infection clearly contributes to the functional decline of the immune responsiveness with age.

## 5. Vaccine-induced antibody responses wane rapidly in the elderly

Some of the wide-reaching effects of immunosenescence have been described in the previous sections, including the impact on the composition of the T cell pool. Evidence suggests that in the elderly, a large proportion of the memory T cells have characteristics of replicative senescence [33]. The potential far-reaching effects of the presence of senescent T cells is illustrated by the correlation between poor response to vaccination in the elderly and an increase in the proportion of CD8^+ ^T cells that lack expression of CD28 [34-36].

The underlying cause for the putative occurrence of replicative senescence within the T cell memory pool during aging may relate to the exquisite specificity of each lymphocyte. Although it is estimated that an individual CD4^+ ^T cell can respond to approximately 3 × 10^5 ^different 11-mer peptides [37], there is an enormous repertoire of immune cells and the number of cells that can recognise and respond to any single antigen may be extremely small. Thus, to generate a sufficient quantity of specific effector cells to fight an infection, an activated lymphocyte must proliferate extensively before its progeny differentiate into effector cells. For this reason, a limitation on the process of cell division could potentially have devastating consequences on immune function.

**Figure 4:**
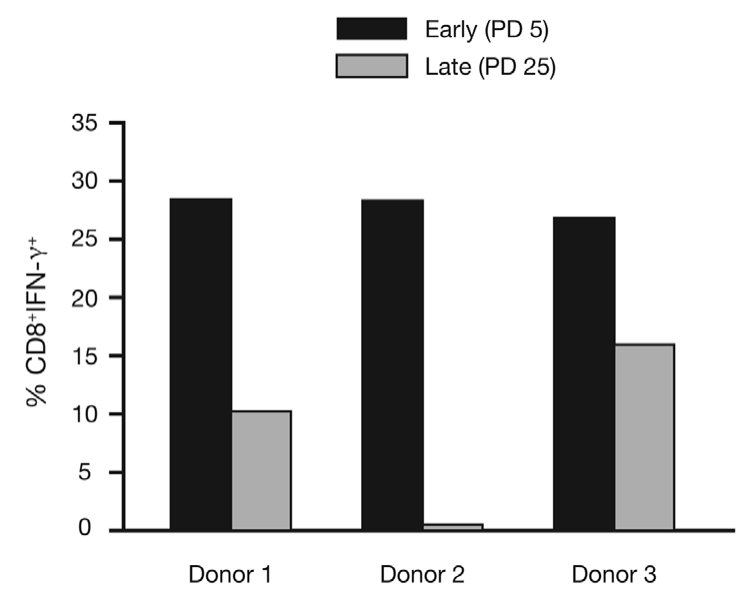
**The decline in interferon-γ (IFN-γ) production with culture age [36]. Reproduced with permission from Blackwell Publishing**. Long-term HIV-specific cultures were established by repeated stimulation of CD8 T-cells from HLA-A2+ HIV-infected persons with the appropriate gag, pol and env peptides. Early passage (5 population doublings, PD 5) and late passage (PD 25) cultures from three different donors were assessed for intracellular IFN-γ using flow cytometry.

In situations of repeated interaction with an antigen over an extended period, as occurs during chronic infections, the relevant T cells undergo extensive proliferation, as indicated by telomere length measurements. Studies that model this process in cell culture using an allogeneic lymphoblastoid cell line as antigen have shown that extensive antigen-driven cell division ultimately triggers an irreversible cell-cycle arrest by a process referred to as replicative senescence. Using this same model, we have shown that cultures of senescent CD8^+ ^T cells also show resistance to apoptosis, permanent loss of CD28 expression, reduced ability to respond to stress, and an altered cytokine profile. If similar changes in cytokine profiles occur *in vivo*, T cell communication and response to vaccination may be affected. An example of this alteration in cytokine profiles is the increase in the production of tumour necrosis factor alpha (TNF-α) and IL-6 [36], changes that occur in cell culture, and which mirror cytokine profiles observed in the frail elderly. Conversely, reduced production of antiviral cytokine, interferon-γ, was observed in cultures of HIV-specific senescent CD8^+ ^T cells established from HIV+ donors (Figure 4). HIV disease shows multiple features of premature immunological aging [37].

A further change observed in senescent CD8^+ ^T cells is the critically short telomere length [36]. Telomerase, an enzyme that is upregulated in activated T cells, re-elongates telomeres *in vivo *and the decline in the activity of this protein is thought to play a key role in replicative senescence. CD8^+ ^T cells that are repeatedly stimulated with antigen in cell culture lose the ability to activate telomerase [38,39]. Gene therapy using the catalytic human telomerase (hTERT), improves the function of these cells [40]. These proof-of-principle studies suggest that telomerase-based approaches, either genetic or pharmacologic, may retard or prevent CD8^+^T cell replicative senescence.

Several factors may drive T cell senescence *in vivo*, among which are infections with viruses that establish latency, such as CMV or Epstein-Barr virus (EBV). These viruses can chronically stimulate T cells, and may be responsible for the presence of clonal expansions of virus-specific CD8^+ ^T cells in the elderly [32]. Thus, in addition to telomerase-based strategies for preventing or reversing this senescence, physical removal of senescent cells has been proposed [41]. If senescence can be prevented or reversed, either using telomerase-based approaches or physical intervention, this would improve the immune response and the effectiveness of vaccination in the elderly.

## 6. Conclusions

The evidence presented here demonstrates the impact of immunosenescence on multiple aspects of the immune system. Examples of some clinical outcomes of this age-related decline in immune function are the increased risk of elderly persons to succumb to infections and their compromised response to vaccination. However, despite the reduced response, vaccination can provide valuable protection for the elderly as prevention of diseases such as influenza, which causes significant morbidity and mortality in this population, is more effective than treatment [4]. Furthermore, vaccination can also have an important role in the prevention of more serious complications, for example pneumonia, cerebrovascular accident, myocardial infarction and other cardiovascular diseases following influenza infection [42,43].

The findings discussed here illustrate the continuing challenges faced in providing effective vaccination coverage against infectious diseases in the elderly. Among these, a particular challenge is represented by vaccination against influenza, due to the very heavy toll of mortality in the elderly every year because of the reduced effectiveness of currently available influenza vaccines in this vulnerable age group. Better vaccine efficacy in the elderly may require a two-pronged attack on the problem, consisting of an improvement in the immune response and an alteration to the vaccine formulation. Reversing the decline in the immune response could be achieved by removing senescent cells, therefore eliminating any potentially detrimental effects emanating from these cells, and replacing these cells with naïve cells through increased thymic output. There are several potential approaches to reversing thymic atrophy and increasing the number of recent thymic emigrants but very few for removing senescent cells. The second prong of the assault on the problem would be to produce a more effective vaccine. Several strategies have been explored, including the use of high dose vaccines [44,45], DNA vaccines with an immunostimulatory patch [46], virosomal vaccines [47] and adjuvanted vaccines [48,49]. Adjuvanted influenza vaccines, such as those containing the oil-in-water emulsion MF59™ and possibly others, when available in the future, have an important role to play in the vaccination of vulnerable populations. Indeed, adjuvanted influenza vaccines have been shown to induce stronger and more effective serologic response in the elderly than conventional non-adjuvanted vaccines, not only against homologous but also against heterovariant strains [48,49]. Adjuvanted vaccines strongly support the notion that better vaccines can be designed with the aim of overcoming immunosenescence and/or improving protection in the elderly population.

## Competing interests

Giuseppe Del Giudice is an employee of Novartis Vaccines, which has provided editorial support for this review; none of the other authors have received financial remuneration for this work or have competing interests.

## Authors' contributions

All authors contributed equally to this review article.
